# Evidence against a Role for the JIL-1 Kinase in H3S28 Phosphorylation and 14-3-3 Recruitment to Active Genes in *Drosophila*

**DOI:** 10.1371/journal.pone.0062484

**Published:** 2013-04-30

**Authors:** Chao Wang, Changfu Yao, Yeran Li, Weili Cai, Xiaomin Bao, Jack Girton, Jørgen Johansen, Kristen M. Johansen

**Affiliations:** Department of Biochemistry, Biophysics, and Molecular Biology, Iowa State University, Ames, Iowa, United States of America; CNRS UMR7275, France

## Abstract

JIL-1 is the major kinase controlling phosphorylation of histone H3S10 and has been demonstrated to function to counteract heterochromatization and gene silencing. However, an alternative model has been proposed in which JIL-1 is required for transcription to occur, additionally phosphorylates H3S28, and recruits 14-3-3 to active genes. Since these findings are incompatible with our previous demonstration that there are robust levels of transcription in the complete absence of JIL-1 and that JIL-1 is not present at developmental or heat shock-induced polytene chromosome puffs, we have reexamined JIL-1’s possible role in H3S28 phosphorylation and 14-3-3 recruitment. Using two different H3S28ph antibodies we show by immunocytochemistry and immunoblotting that in *Drosophila* the H3S28ph mark is not present at detectable levels above background on polytene chromosomes at interphase but only on chromosomes at pro-, meta-, and anaphase during cell division in S2 cells and third instar larval neuroblasts. Moreover, this mitotic H3S28ph signal is also present in a *JIL-1* null mutant background at undiminished levels suggesting that JIL-1 is not the mitotic H3S28ph kinase. We also demonstrate that H3S28ph is not enriched at heat shock puffs. Using two different pan-specific 14-3-3 antibodies as well as an enhancer trap 14-3-3ε-GFP line we show that 14-3-3, while present in salivary gland nuclei, does not localize to chromosomes but only to the nuclear matrix surrounding the chromosomes. In our hands 14-3-3 is not recruited to developmental or heat shock puffs. Furthermore, using a *lacO* repeat tethering system to target LacI-JIL-1 to ectopic sites on polytene chromosomes we show that only H3S10ph is present and upregulated at such sites, not H3S28ph or 14-3-3. Thus, our results argue strongly against a model where JIL-1 is required for H3S28 phosphorylation and 14-3-3 recruitment at active genes.

## Introduction

The JIL-1 kinase localizes specifically to euchromatic interband regions of polytene chromosomes and is the kinase responsible for histone H3S10 phosphorylation at interphase in *Drosophila* [1], [2]. Furthermore, JIL-1 is enriched about two-fold on the male X chromosome and implicated in transcriptional regulation [1]. In a recent study Cai et al. [3] determined the genome-wide relationship of JIL-1 kinase mediated H3S10 phosphorylation with gene expression. Interestingly, the results indicated that nearly as many genes were activated as repressed in the absence of the epigenetic H3S10ph mark. Furthermore, Wang et al. [4] provided evidence that gene expression levels at the *white* locus were directly correlated with the levels of the H3K9me2 mark independently of the state of the H3S10ph mark, which was not required for either transcription or gene activation to occur. Thus, these findings taken together with previous studies suggested a model where H3S10 phosphorylation functions to indirectly regulate transcription by counteracting H3K9 dimethylation and gene silencing in a finely tuned balance [3]–[8]. However, an alternative scenario has been proposed in which JIL-1 is required for transcription to occur, additionally phosphorylates H3S28, and recruits 14-3-3 to active genes [9]–[11]. Since these findings are incompatible with the results of Cai et al. [12] demonstrating that there are robust levels of transcription in the complete absence of JIL-1 and that JIL-1 is not enriched at developmental or heat shock-induced polytene chromosome puffs, in this study we reexamined JIL-1’s possible role in H3S28 phosphorylation and 14-3-3 recruitment. The results suggest that JIL-1 is not a H3S28 kinase and that it is not involved in 14-3-3 recruitment in *Drosophila*.

## Materials and Methods

### *Drosophila Melanogaster* Stocks

Fly stocks were maintained at 25°C according to standard protocols [13] and Canton S was used for wild type preparations. The *JIL-1^z2^* null allele is described in Wang et al. [2] as well as in Zhang et al. [14]. The 14-3-3ε-GFP fly trap line (G00082) was obtained from the Yale Fly Trap Stock Center and verified by PCR amplification and sequencing at the Iowa University Sequencing Facility. The *H2AvDmRFP1* transgenic line was the generous gift of Dr. S. Heidmann and has been previously described [15], [16]. The JIL-1-CTD-CFP construct containing JIL-1 sequence from aa 927–1207 in the *pYES* vector is described in Wang et al. [8]. A *da-GAL4* driver introduced by standard genetic crosses was used to express the transgenes. The *LacI-JIL-1 pUAST* transgenic fly line is described in Deng et al. [17] with expression driven using the *Sgs3-GAL4* driver (obtained from the Bloomington Stock Center) introduced by standard genetic crosses. The Lac operator insertion line P11.3 is described in Li et al. [18] and in Deng et al. [17]. For heat shock experiments, wandering third instar larvae were subjected to 30 min of heat shock treatment at 37°C as described previously [19].

### Immunohistochemistry

Salivary gland nuclei smush preparations were made as described in Wang et al. [2] and standard polytene chromosome squash preparations were performed as in Cai et al. [20] using either 1 or 5 minute fixation protocols and labeled with antibody as described in Jin et al. [1]. Larval brain squashes were performed according to the protocol of Bonaccorsi et al. [21] with minor modifications as described in Ding et al. [22]. S2 cell and whole mount salivary gland immunocytochemistry using 4% Paraformaldehyde fixation protocols were performed as described in Qi et al. [23]. Primary antibodies used in this study include rabbit anti-H3S10ph (Cell Signaling), chicken anti-JIL-1 [24], rabbit anti-H3S28ph (Cell Signaling), rabbit anti-H3S28ph (Millipore-Upstate), goat anti-histone H3 (Santa Cruz), rabbit anti-pan 14-3-3 (Cell Signaling), rabbit anti-pan 14-3-3 (Santa Cruz), mouse anti-lacI (Millipore-Upstate), mouse anti-Pol IIo^ser5^ (Covance), chicken anti-GFP (Aves Lab), rabbit anti-histone H1 (Active Motif), and mouse anti-tubulin (Sigma). DNA was visualized by staining with Hoechst 33258 (Molecular Probes) in PBS. The appropriate species- and isotype- specific Texas Red-, TRITC-, and FITC-conjugated secondary antibodies (Cappel/ICN, Southern Biotech) were used (1∶200 dilution) to visualize primary antibody labeling. All secondary antibodies used were verified to be without non-specific cross-reactivity to polytene squash preparations. The final preparations were mounted in 90% glycerol containing 0.5% n-propyl gallate. The preparations were examined using epifluorescence optics on a Zeiss Axioskop microscope and images were captured and digitized using a cooled Spot CCD camera. Images were imported into Photoshop where they were pseudocolored, image processed, and merged. In some images non-linear adjustments were made to the channel with Hoechst labeling for optimal visualization of chromosomes. For each experimental condition the labeling of chromosomes from at least 48 individual nuclei from a minimum of 8 different larvae were analyzed.

For live imaging third instar larval salivary glands expressing 14-3-3ε-GFP and H2Av-RFP were dissected and mounted in physiological saline (110 mM NaCl, 4 mM KCl, 2 mM CaCl_2_, 10 mM glucose, 10 mM HEPES, pH 7.4) as in Deng et al. [15] and in Yao et al. [25]. In some cases, 25–50% glycerol was added to the physiological saline in order to prevent drift of the preparations. Confocal images of whole mount and live salivary glands were obtained using a Leica confocal TCS SP5 microscope system as previously described [22], [25].

### Immunoblot Analysis

Protein extracts were prepared from dissected third instar larval salivary glands or brains homogenized in a buffer containing: 20 mM Tris-HCl pH 8.0, 150 mM NaCl, 10 mM EDTA, 1 mM EGTA, 0.2% Triton X-100, 0.2% NP-40, 2 mM Na_3_VO_4_, 1 mM PMSF, 1.5 µg/ml aprotinin. Proteins were separated by SDS-PAGE and immunoblotted according to standard procedures [26]. For these experiments we used the Bio-Rad Mini PROTEAN III system, electroblotting to 0.2 µm nitrocellulose, and using anti-mouse, anti-goat or anti-rabbit HRP-conjugated secondary antibody (Bio-Rad) (1∶3000) for visualization of primary antibody. Antibody labeling was visualized using chemiluminescent detection methods (SuperSignal West Pico Chemiluminescent Substrate, Pierce). The immunoblots were digitized using a ChemiDoc-It®TS2 Imager (UVP,LCC). Analysis of immunoblots were based on at least 3 independent replications.

### Preparation of Salivary Gland Nuclear and Cytoplasmic Fractions

Salivary glands from 100 to 200 animals were dissected and homogenized with a Dounce homogenizer (loose pestle) in hypotonic buffer (10 mM Tris-HCl, pH 8.0; 1 mM KCl; 1.5 mM MgCl_2_, 0.5% NP-40, 1 mM DTT, and including the proteinase inhibitors aprotinin, pepstatin, and PMSF). The resulting lysate was incubated on ice for 20–30 min before nuclei were pelleted by centrifugation at 3000 rpm for 5 min at 4°C and the supernatant collected as the cytoplasmic fraction. Subsequently, the pellet was washed twice with 1 ml of hypotonic buffer and pelleted at 2000 and 6000 rpm, respectively. The pelleted nuclei were lysed in Nuclei Extract Buffer containing: 20 mM Hepes pH 7.4, 10% glycerol, 350 mM NaCl, 0.1% Triton, 1 mM PMSF, and 1 mM DTT on ice for 30 min. After centrifugation at 18,000 rpm for 10 min, the supernatant was collected as the nuclear fraction. The nuclear and cytoplasmic fractions were analyzed by SDS-PAGE and immunoblotting as described above. The fractionation was replicated 3 times.

## Results

### JIL-1 is not a Histone H3S28 Kinase in *Drosophila*

A recent study by Kellner et al. [11] using immunocytochemistry claimed that the H3S28ph mark is present at interbands of polytene chromosomes, that it accumulates at heat shock puffs, and that the JIL-1 kinase is responsible for this H3S28 phosphorylation. The JIL-1 kinase localizes to interband regions of polytene chromosomes and is upregulated about two-fold on the male X chromosome [1], [2]. Consequently, potential interphase histone H3S28 phosphorylation mediated by JIL-1 would be expected to show co-localization with JIL-1, show upregulation on the male X chromosome, and labeling should be absent or reduced in homozygous *JIL-1* (*JIL-1^z2^/JIL-1^z2^*) null nuclei. To test whether this is the case we performed immunolabeling of polytene squash preparations from wild-type and *JIL-1* null third instar larvae using two different commercially available H3S28ph antibodies from Upstate/Millipore (UP) and Cell Signaling (CS), respectively. The H3S28ph (UP) antibody is the same antibody used in the study of Kellner et al. [11].

**Figure 1:**
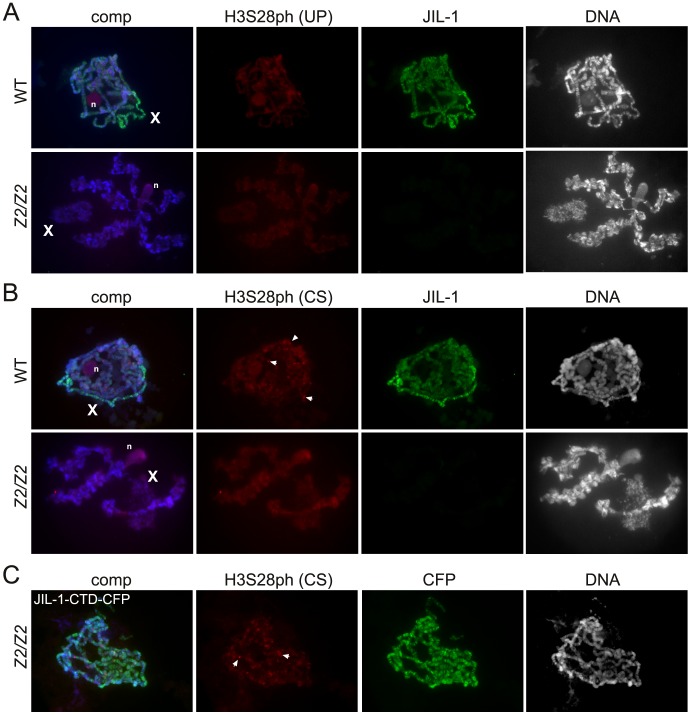
Polytene squash preparations from male wild-type and ***JIL-1*** **null salivary glands labeled with H3S28ph antibodies.** (A) Wild-type and homozygous *JIL-1^z2^/JIL-1^z2^* null (*z2/z2*) squash preparations labeled with H3S28ph (UP) antibody (in red), JIL-1 antibody (in green), and Hoechst (DNA, in blue/gray). (B) Wild-type and *JIL-1^z2^/JIL-1^z2^* null (*z2/z2*) squash preparations labeled with H3S28ph (CS) antibody (in red), JIL-1 antibody (in green), and Hoechst (DNA, in blue/gray). (C) Polytene squash preparation from a *JIL-1^z2^/JIL-1^z2^* null (*z2/z2*) salivary gland expressing a CFP-tagged JIL-1-CTD construct (JIL-1-CTD-CFP) labeled with H3S28ph (CS) antibody (in red); GFP/CFP antibody (in green), and Hoechst (DNA, in blue/gray). The male X chromosome is indicated by an X and the nucleolus by an n. Examples of interband labeling by the H3S28ph (CS) antibody are indicated by arrowheads in (B) and (C).

**Figure 2:**
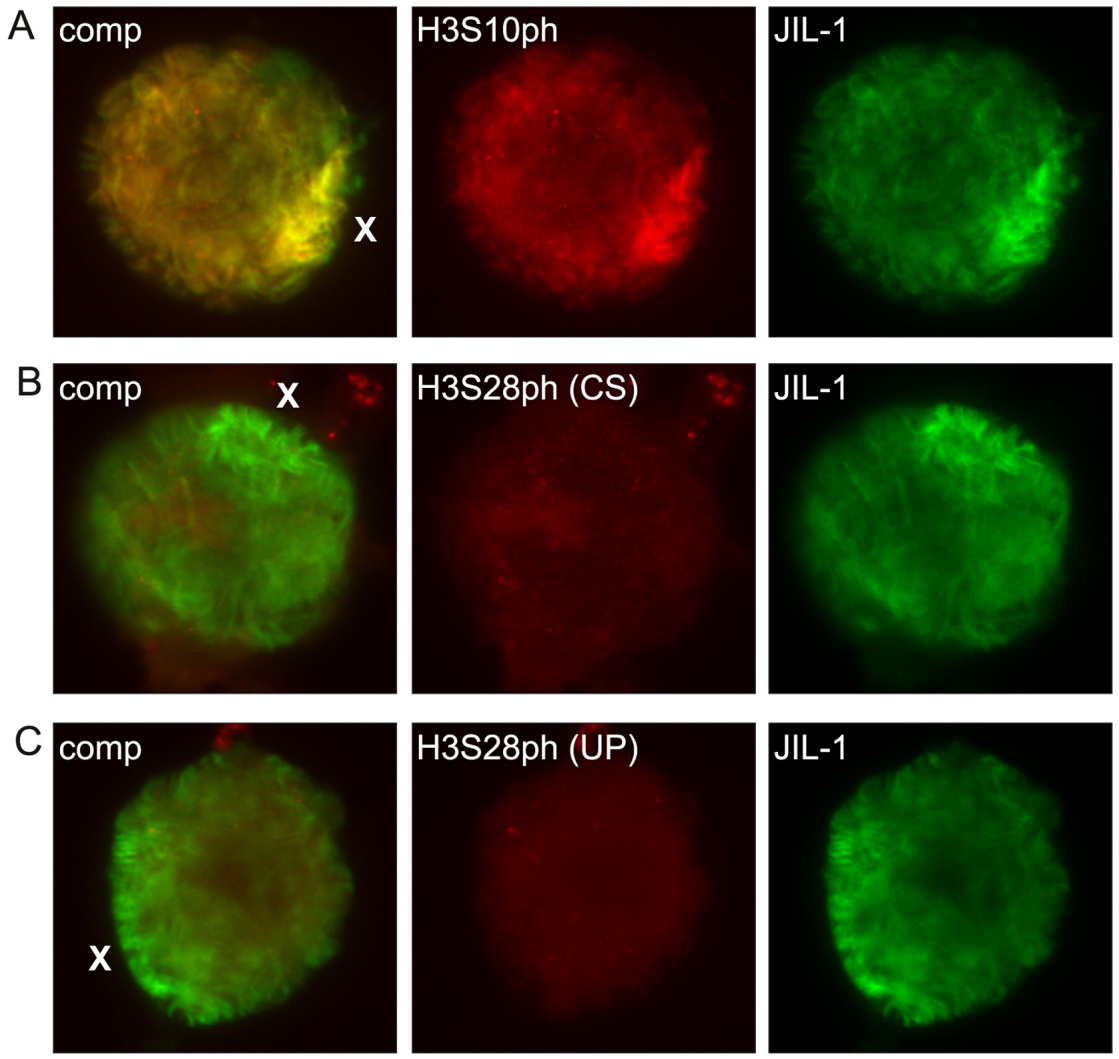
Histone H3S10ph and H3S28ph antibody labelings of male salivary gland nuclei smush preparations. (A) Double labeling with H3S10ph antibody (in red) and JIL-1 antibody (in green) demonstrating co-localization and the characteristic upregulation of JIL-1 and H3S10ph labeling on the male X chromosome (X). (B) Double labeling with H3S28ph (CS) antibody (in red) and JIL-1 antibody (in green). (C) Double labeling with H3S28ph (UP) antibody (in red) and JIL-1 antibody (in green). In contrast to the labeling of the H3S10ph antibody there was no discernable labeling above background of the autosomes or the male X chromosome by either of the two H3S28ph antibodies.

Figure 1 shows examples of polytene squash preparations double labeled with H3S28ph and JIL-1 antibody. We detected no or little specific labeling above background of either H3S28ph antibody. Under such conditions it is difficult to determine what the correct exposure of the labeling (or non-labeling) should be. Therefore, in Fig. 1 the H3S28ph images were adjusted such that background labeling of the nucleolus is just visible. As illustrated in Fig. 1A the H3S28ph (UP) level of labeling on the chromosome arms was no different from that of the nucleolus as well as that of the chromosomes in the *JIL-1* null background with no discernable banding pattern. However, the H3S28ph (CS) antibody did label a number of discrete bands on the chromosome arms above background levels although these bands did not overlap with any JIL-1 labeling (Fig. 1B). While difficult to discern because of the grossly perturbed chromosome morphology [15] such discrete labeling was also present in the *JIL-1* null background (Fig. 1B). Thus, in order to verify this we expressed a CFP-tagged JIL-1 carboxy-terminal construct (JIL-1-CTD-CFP) in the *JIL-1* null background that rescues chromosome morphology to near wild-type without JIL-1 kinase activity [27]. As illustrated in Fig. 1C discrete bands similar to those of wild-type preparations were clearly present under these conditions indicating that they were not caused by JIL-1 kinase activity. Moreover, there was no discernable labeling upregulated on the male X chromosome coincident with JIL-1 labeling by either H3S28ph antibody (Fig. 1). Thus, in standard polytene squash preparations we could detect no specific H3S28ph antibody labeling correlated with JIL-1 kinase activity. However, as previously reported [2], [12] the highly acidic fixation conditions of conventional squash protocols may prevent reliable antibody labeling of some phospho-epitopes. For example, in such preparations H3S10ph antibody labeling can be extremely weak and except for rare cases the upregulation of H3S10 phosphorylation on the male X chromosome [2], [12] cannot be detected indicating incomplete or defective antibody recognition. Thus, in this study, to overcome such potential difficulties we labeled “smush” preparations with the two H3S28ph antibodies as well as with H3S10ph antibody for comparison (Fig. 2). The smush preparation is a modified whole-mount staining technique where nuclei from dissected salivary glands are gently compressed beneath a coverslip to flatten them before fixation in a standard paraformaldehyde/PBS solution with a physiological pH [2], [12]. As illustrated in Fig. 2A in such preparations there were extensive overlap between H3S10ph and JIL-1 labeling including upregulation on the male X chromosome whereas there was no discernable labeling above background by either H3S28ph antibody (Figs 2B, 2C).

**Figure 3:**
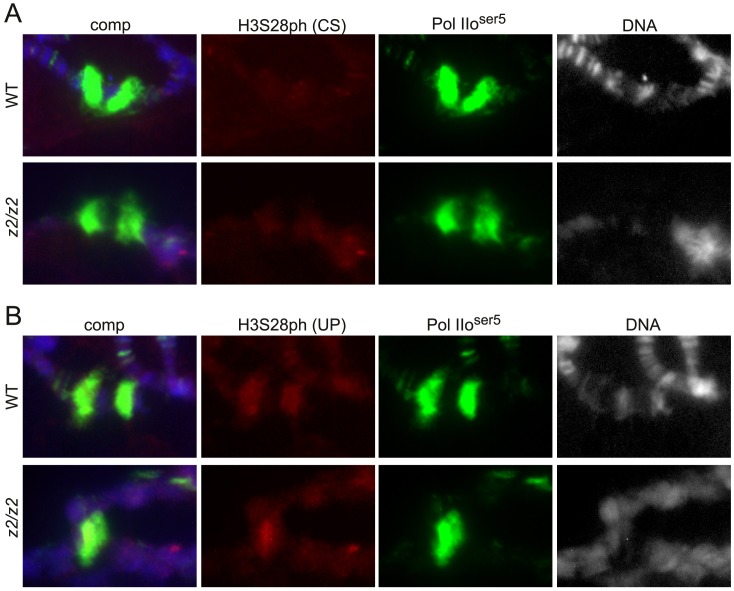
Polytene chromosomes from wild-type and *JIL-1* null salivary glands labeled with two different H3S28ph antibodies after heat shock treatment. (A) Wild-type and homozygous *JIL-1^z2^/JIL-1^z2^* null (*z2/z2*) squash preparations labeled with H3S28ph (CS) antibody (in red), Pol IIo^ser5^ antibody (in green), and Hoechst (DNA, in blue/gray). There was no obvious labeling by the antibody above background of the heat shock puffs although they were robustly labeled by the Pol IIo^ser5^ antibody. (B) Wild-type and homozygous *JIL-1^z2^/JIL-1^z2^* null (*z2/z2*) squash preparations labeled with H3S28ph (UP) antibody (in red), Pol IIo^ser5^ antibody (in green), and Hoechst (DNA, in blue/gray). This antibody weakly labeled heat shock puffs above background levels; however, such labeling was also observed in the *JIL-1* null mutant background.

**Figure 4:**
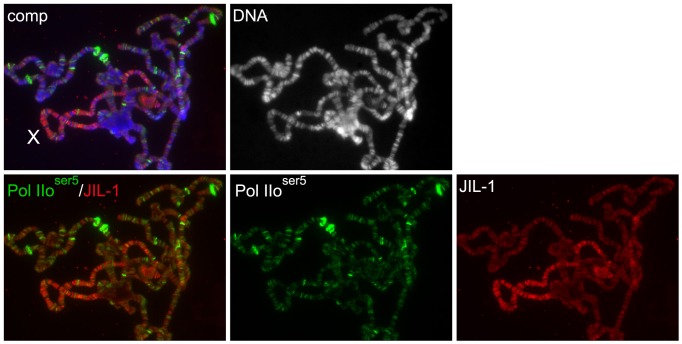
JIL-1 does not co-localize with the paused form of RNA Polymerase II and is not upregulated at developmental puffs. The polytene chromosome squash preparation from a wild type larvae was triple labeled with Pol IIo^ser5^ antibody (in green), JIL-1 antibody (in red), and Hoechst (DNA, gray/blue). At many sites that showed especially high levels of Pol IIo^ser5^ staining such as at developmental puffs, there was little or no JIL-1 antibody labeling. The male X chromosome is indicated by an X.

In order to compare the potential distribution of H3S28ph at heat shock puffs in wild-type and *JIL-1* null mutant backgrounds we double labeled polytene chromosome squash preparations with either of the two H3S28ph antibodies and with antibody to the paused form of RNA Polymerase II (Pol IIo^ser5^), that serves as a marker for heat shock puff regions [9]. In response to heat shock treatment there is a redistribution of Pol IIo^ser5^ labeling which is reduced at most sites, while being dramatically upregulated at heat shock puffs where transcription of heat shock-activated genes is occuring [9], [12]. As illustrated in Fig. 3A there was no obvious labeling by the H3S28ph (CS) antibody above background of the 87A/C heat shock puffs although they were robustly labeled by the Pol IIo^ser5^ antibody. In contrast, we found that the H3S28ph (UP) antibody in some but not all preparations weakly labeled the 87A/C puffs above background after heat shock (Fig. 3B, upper panel). However, such labeling of heat shock puffs could also be found in polytene chromosome squashes from *JIL-1* null mutant larvae that are devoid of JIL-1 kinase activity (Fig. 3B, lower panel). Thus, we conclude that the labeling of heat shock puffs by this antibody is due to non-specific cross-reactivity possibly with proteins involved in the heat shock response. It should be noted that at least one H3S10ph antibody has been demonstrated to have similar non-specific cross-reactivity with heat shock puffs [12]. In addition, it should be noted that there is little or no overlap between JIL-1 and Pol IIo^ser5^ antibody labeling of polytene squash preparations including developmental puffs (Fig. 4) as also previously reported for JIL-1 and the elongating form of RNA Polymerase II (Pol IIo^ser2^) [12].

**Figure 5:**
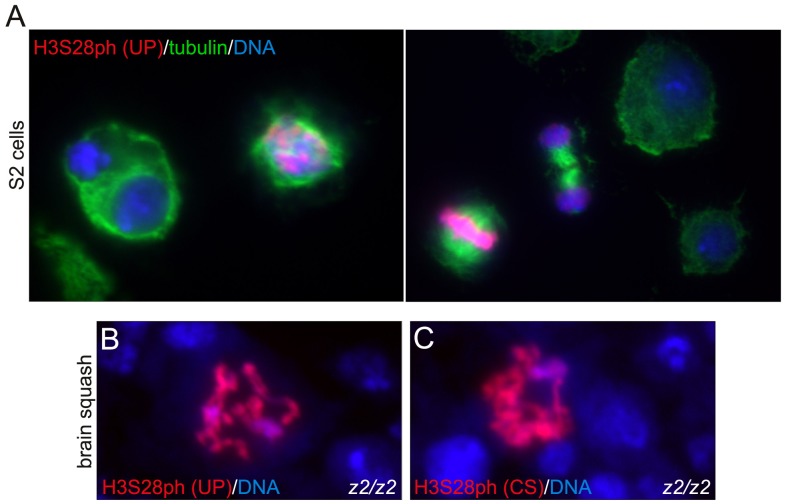
H3S28 phosphorylation of mitotic chromosomes. (A) S2 cell preparations labeled with H3S28ph (UP) antibody (in red), tubulin antibody (in green), and Hoechst (DNA in blue). The images show clear H3S28ph antibody labeling of chromosomes at pro- and metaphase, but little or no labeling of interphase nuclei. (B–C) Squash preparations from homozygous *JIL-1^z2^/JIL-1^z2^* null (*z2/z2*) third instar larval brains labeled with H3S28ph (UP) or H3S28ph (CS) antibody (in red) and with Hoechst (DNA in blue). Mitotic chromosomes were robustly labeled by both antibodies.

In higher organisms H3S28 phosphorylation serves mainly as a mitotic marker (reviewed in [28]). In order to determine whether this is also the case in *Drosophila* we triple labeled S2 cell preparations with H3S28ph and tubulin antibodies and with Hoechst. As illustrated in Fig. 5A there was no or little labeling above background by the H3S28 (UP) antibody of S2 cells at interphase. However, there was clear labeling of chromosomes at pro-, meta-, and anaphase of S2 cells undergoing cell division. Similar results were obtained with the H3S28 (CS) antibody (data not shown). These results also serve to demonstrate that the antibodies in our hands are functional and indeed recognize the H3S28ph epitope. Figure 5B and 5C demonstrate that metaphase chromosomes from *JIL-1* null mutant third instar larval brain squashes were robustly labeled by both the H3S28 (UP) and H3S28 (CS) antibodies indicating that JIL-1 is not the mitotic H3S28ph kinase in *Drosophila*.

**Figure 6:**
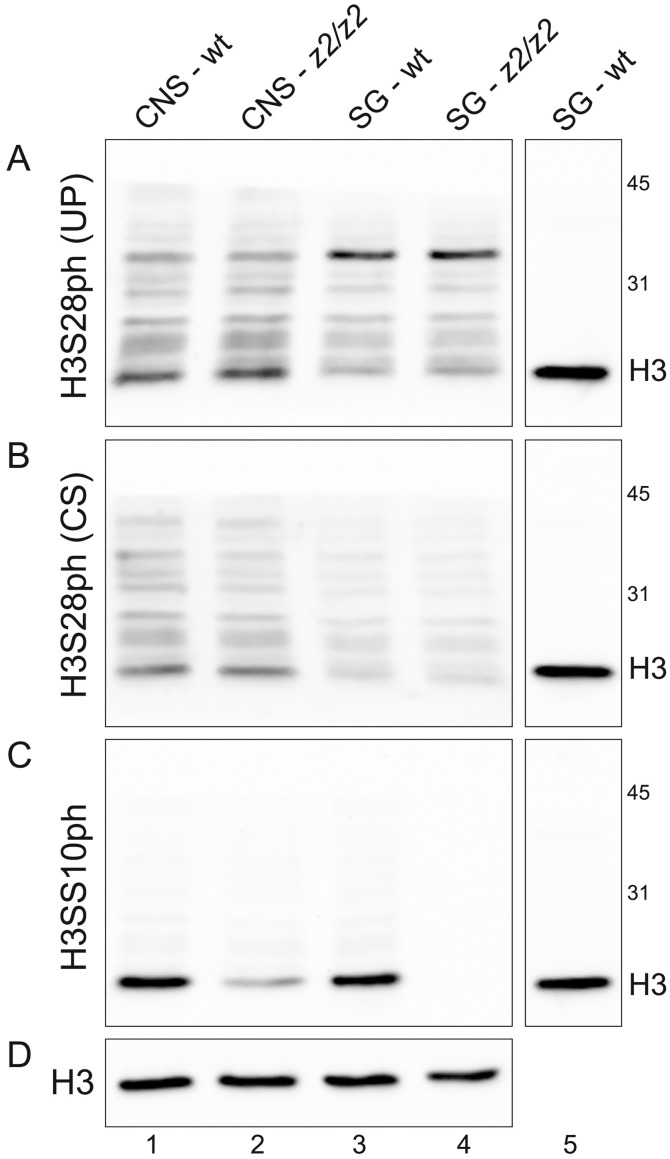
Immunoblot characterization of two different H3S28ph antibodies. (A–D) Immunoblots of protein extracts from salivary glands (SG) or the CNS from wild-type (wt) or *JIL-1^z2^/JIL-1^z2^* (*z2/z2*) larvae labeled with H3S28ph (UP) antibody (A), H3S28ph (CS) antibody (B), and H3S10ph antibody (C). Labeling with histone H3 (H3) antibody was used as a loading control (D) and as a marker for the relative migration of histone H3 (lane 6). The relative migration of molecular size markers is indicated in kD.

To further validate the results obtained by immunocytochemistry we performed immunoblot analysis of protein extracts from wild-type and *JIL-1* null mutant salivary glands and larval brains. Salivary gland cells are strictly at interphase whereas third instar larval brains contain a significant population of mitotic nuclei (>5%). As illustrated in Fig. 6 there was no difference in the patterning of labeling of protein extracts from wild-type and *JIL-1* null salivary glands separated by SDS-PAGE and immunoblotted with H3S28ph (UP) (Fig. 6A) and H3S28ph (CS) (Fig. 6B) antibody, respectively. However, at the position of the histone H3 marker there was a discernable increase in both the wild-type and *JIL-1* null mutant CNS lanes of the labeling by both antibodies. These data support the conclusions from the immunocytochemical studies that there is no or little H3S28 phosphorylation at interphase, only at mitosis, and that JIL-1 does not contribute to H3S28 phosphorylation. Protein bands labeled by the antibodies migrating above the histone H3 are likely to be caused by non-specific cross-reactivity. For comparison Fig. 6C shows the labeling pattern for H3S10 phosphorylation. There is comparable labeling by the antibody of wild-type CNS and salivary gland extracts; however, this labeling is completely abolished in *JIL-1* null mutant salivary glands, whereas some labeling remains in *JIL-1* null mutant CNS extracts due to mitotic H3S10 phosphorylation by the Aurora B kinase [2], [29]. Thus, these results confirm that JIL-1 is the interphase H3S10ph kinase and strongly indicate that JIL-1 has no H3S28 phosphorylation activity at any point in the cell cycle.

### 14-3-3 does not Bind to Chromosomes or Colocalize with JIL-1 in *Drosophila* Salivary Gland Cells

In a recent study Karam et al. [10] claimed that the small scaffolding protein 14-3-3 (reviewed in [30]) binds to polytene chromosomes and is recruited to active genes in a JIL-1 and H3S10 phosphorylation dependent manner. In an attempt to reproduce these results we performed double labeling studies with JIL-1 and 14-3-3 antibodies of polytene chromosome squash preparations. For these experiments we used two commercially available pan-specific 14-3-3 antibodies from Santa Cruz Biotechnology (SZ) and Cell Signaling (CS), respectively. In *Drosophila*, there are two 14-3-3 isoforms (ε and ζ) which share a highly conserved amino-terminal amino acid stretch to which the antibodies were made [10]. The 14-3-3 (SZ) antibody is the same antibody used in the study of Karam et al. [10].

**Figure 7:**
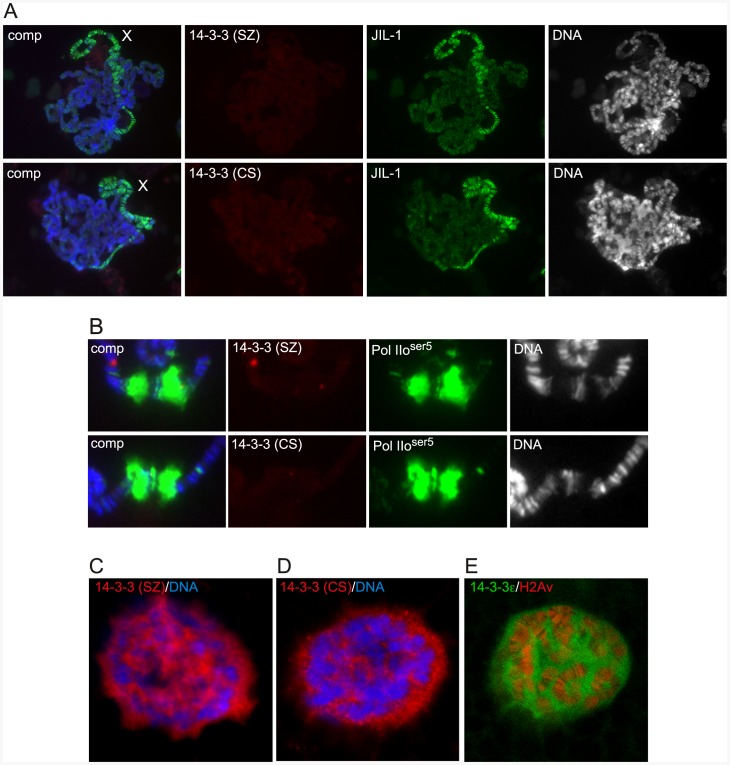
14-3-3 antibody labeling of salivary gland chromosomes and nuclei. (A–B) 14-3-3 antibody labeling of male polytene squash preparations before and after heat shock treatment. (A) Wild-type squash preparations labeled with 14-3-3 (SZ) or 14-3-3 (CS) antibody (in red), JIL-1 antibody (in green), and Hoechst (DNA, in blue/gray). (B) Wild-type squash preparations after heat shock treatment labeled with 14-3-3 (SZ) or 14-3-3 (CS) antibody (in red), Pol IIo^ser5^ antibody (in green), and Hoechst (DNA, in blue/gray). No or little specific labeling above background of either 14-3-3 antibody was discernable. (C–E) 14-3-3 localizes to the nuclear matrix surrounding the chromosomes. (C–D) Confocal sections of whole-mount salivary gland nuclei labeled with 14-3-3 (SZ) or 14-3-3 (CS) antibody (in red) and Hoechst (DNA in blue). (E) Confocal section of a live salivary gland nuclei from a 14-3-3ε-GFP (in green) enhancer trap line co-expressing histone H2Av-RFP (in red).

Figure 7A shows examples of polytene squash preparations double labeled with 14-3-3 and JIL-1 antibody. We observed no or little specific labeling above background of either 14-3-3 antibody as well as no discernable labeling upregulated on the male X chromosome coincident with JIL-1 labeling (Fig. 7A). Thus, in standard polytene squash preparations we could detect no specific 14-3-3 antibody labeling correlated with JIL-1 localization. Furthermore, in order to compare the potential recruitment of 14-3-3 at heat shock puffs we double labeled polytene chromosome squash preparations with 14-3-3 antibody and with antibody to the heat shock puff marker Pol IIo^ser5^ after heat shock. As illustrated in Fig. 7B there was no obvious labeling by either 14-3-3 antibody above background of the 87A/C heat shock puffs although they were robustly labeled by the Pol IIo^ser5^ antibody suggesting that 14-3-3 is not recruited to actively transcribed chromatin regions.

**Figure 8:**
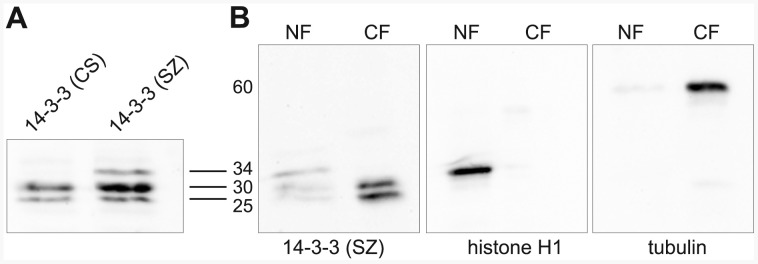
Immunoblot characterization of two different 14-3-3 antibodies. (A) Immunoblot of protein extracts from salivary glands labeled with 14-3-3 (CS) or 14-3-3 (SZ) antibody. (B) Immunoblots of the relative distribution of 14-3-3 proteins in nuclear (NF) and cytoplasmic (CF) fractions (left panel) probed with the 14-3-3 (SZ) antibody. The quality of the fractionation was verified by labeling of the fractions with histone H1 antibody (middle panel) and tubulin antibody (right panel). The relative migration of the proteins based on the migration of molecular size markers is indicated in kD.

14-3-3 has been reported to be present in the cytoplasm as well as in the nucleus [31], [32] and to further explore its localization in salivary gland cells we double labeled fixed whole mount preparations of dissected salivary glands with 14-3-3 antibody and with Hoechst, a marker for DNA. The results obtained were identical with both 14-3-3 antibodies and revealed nuclear as well as cytoplasmic 14-3-3 localization. Interestingly, the nuclear localization of 14-3-3 as illustrated in the confocal sections in Figs. 7C and 7D was similar to that of the nuclear matrix proteins Megator and EAST [16], [33], [34] which are present not on the chromosomes but in the nuclear space surrounding the chromosomes. To further verify these results we obtained confocal sections of live salivary gland nuclei from a 14-3-3ε-GFP enhancer trap line co-expressing histone H2Av-RFP. As illustrated in Fig. 7E the 14-3-3ε localization obtained from live imaging was indistinguishable from that of 14-3-3 antibody labeled fixed whole mount preparations confirming these results. On immunoblots of salivary gland protein extracts both 14-3-3 antibodies recognized bands of 25 and 30 kD (Fig. 8A) likely corresponding to the ζ and ε isoforms, respectively [10]. In addition, the 14-3-3 (SZ) antibody recognized a 34 kD band (Fig. 8A). We speculate that this band represents either non-specific cross-reactivity or an alternative 14-3-3 splice form not recognized by the 14-3-3 (CS) antibody. To explore whether the 14-3-3 isoforms had differential distribution in the nucleus and cytoplasm we prepared nuclear and cytoplasmic protein fractions from dissected salivary glands. As illustrated in Fig. 8B the 25 and 30 kD 14-3-3 bands were present in both the nuclear and cytoplasmic fractions whereas the 34 kD band was found only in the nuclear fraction. Thus, these results provide further evidence that 14-3-3ε as well as 14-3-3ζ has both nuclear and cytoplasmic localization in salivary gland cells.

### H3S10ph, but Neither H3S28ph nor 14-3-3, is Upregulated at LacI-JIL-1 Targeting Sites

**Figure 9:**
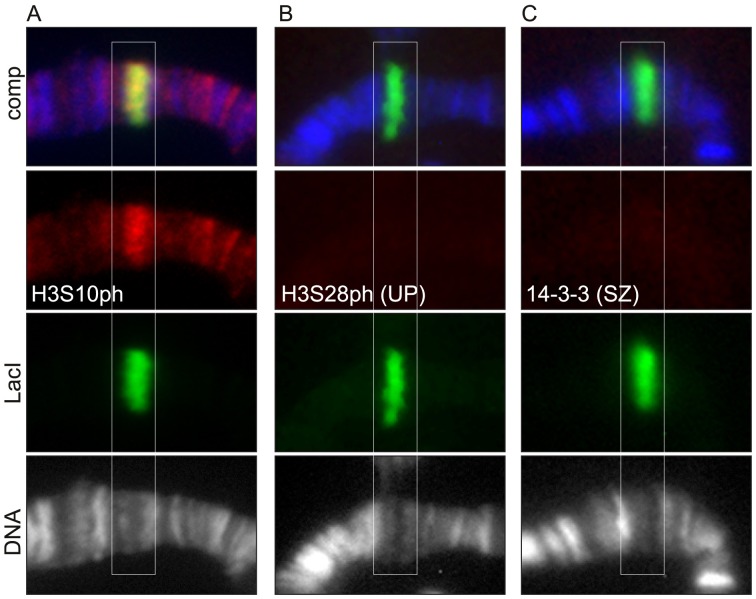
Tethering of LacI-JIL-1 is not associated with upregulation of either H3S28ph or 14-3-3 at the *LacO* insertion site. (A–C) Triple labelings with LacI antibody (in green), H3S10ph antibody (A) or H3S28ph antibody (UP) (B) or 14-3-3 (SZ) antibody (C) (in red), and Hoechst (DNA in blue/gray) of polytene squash preparations from larvae homozygous for the *lacO* repeat line P11.3. There is robust labeling by the H3S10ph antibody at the insertion site; however, there was no discernable signal above background levels when the preparations were labeld with either H3S28ph or 14-3-3 antibody.

We have previously shown that ectopic targeting of JIL-1 using a LacI-tethering system induces robust histone H3S10 phosphorylation and a change in higher order chromatin structure from a condensed heterochromatin-like state to a more open euchromatic state [17]. Therefore, we used this experimental paradigm to test whether such targeting of JIL-1 could induce H3S28 phosphorylation and/or recruitment of 14-3-3. As illustrated in Fig. 9A tethering of LacI-JIL-1 to *lacO* repeats inserted into the middle of a polytene chromosome band in region 96C1-2 resulted in ectopic H3S10 phosphorylation and “opening” of the band as previously reported [17]. However, labeling of the insertion site with H3S28ph (UP) (Fig. 9B) and 14-3-3 (SZ) antibody (Fig. 9C), respectively, revealed no signal above background levels further supporting the conclusion that JIL-1 is not a H3S28 kinase and does not recruit 14-3-3.

## Discussion

In this study we have revisited the possible role of JIL-1 as a H3S28 kinase as well as its relationship to 14-3-3 recruitment. Using two different H3S28ph antibodies we show by immunocytochemistry and immunoblotting that in *Drosophila* the H3S28ph mark is not present at detectable levels above background on polytene chromosomes at interphase but only on chromosomes at pro-, meta-, and anaphase during cell division in S2 cells and third instar larval neuroblasts. Moreover, this mitotic H3S28ph signal is also present in the *JIL-1* null mutant at undiminished levels suggesting that JIL-1 is not the mitotic H3S28ph kinase. We also demonstrate that H3S28ph is not enriched at heat shock puffs. The conclusion that JIL-1 is not a H3S28 kinase is further supported by in vitro phosphorylation experiments where JIL-1 was found to be unable to phosphorylate H3S28, but efficiently phosphorylated H3S10 [35]. Using two different pan-specific 14-3-3 antibodies as well as an enhancer trap 14-3-3ε-GFP line we show that 14-3-3, while present in salivary gland nuclei, does not localize to chromosomes but only to the nuclear matrix surrounding the chromosomes. In our hands 14-3-3 is not recruited to developmental or heat shock puffs. In another study, evidence has been presented that 14-3-3 functions in the heat shock response as a stress induced molecular chaperone that dissolves thermal-aggregated proteins in the cytosol [32]. Furthermore, using a LacI-JIL-1 targeting system to ectopic sites on polytene chromosomes we show that only H3S10ph is present and upregulated at such sites, not H3S28ph or 14-3-3. These results are contrary to those reported previously [9]–[11]. We cannot explain the discrepancies apart from that they may be caused by technical issues and the use in some experiments of cell lines with a significant mitotic population [11]. Furthermore, the conclusions of the present study in contrast to the studies of Kellner et al. [11] and Karam et al. [10] are fully consistent with the demonstration by Cai et al. [12] that neither JIL-1 nor H3S10ph are detectable on developmental or heat shock induced puff regions and that there is little or no overlap between the distribution of JIL-1 and markers for both the paused and elongating forms of RNA Polymerase II [12,35, this study].

The model by Corces and colleagues [10]–[11] that phosphorylation of H3S10 and H3S28 by JIL-1 and consequent recruitment of 14-3-3 and the elongator protein 3 is required to mediate transcription elongation in *Drosophila* has also recently been challenged by analyses of the genome-wide relationship between JIL-1 and transcription [3], [35]. In one of these studies in order to explore the effect of JIL-1 and the epigenetic H3S10 mark on gene expression, Cai et al. [3] compared the global changes in gene expression in wild-type and *JIL-1* null salivary glands. Salivary glands are post-mitotic allowing the comparison of the binding sites of JIL-1 with the locations of the H3S10ph mark. Strikingly, the results showed that of 1,737 genes where expression levels changed at least two-fold in the mutant, a substantial number (39%) of these genes were upregulated whereas 61% were downregulated. Regnard et al. [35] using a RNAi knockdown approach in SL2 cells also observed an increase in transcription of genes after JIL-1 depletion. Furthermore, a study by Wang et al. [4] exploring the relationship between PEV and the relative levels of the H3S10ph and H3K9me2 marks at the *white* gene in wild-type and *w^m4^* backgrounds showed that downregulation of *white* expression in the mutant and absence of H3S10 phosphorylation was correlated with increased levels of the H3K9me2 mark whereas upregulation of the gene was correlated with diminished H3K9 dimethylation. These results are compatible with a model where gene expression levels are modulated by the levels of the H3K9me2 mark independently of the state of the H3S10ph mark, which itself is not required for either transcription or gene activation to occur. Rather, JIL-1 and H3S10 phosphorylation function to indirectly maintain active transcription by counteracting increased levels of H3K9 dimethylation and gene silencing [4], [8], [35].
